# 2 DOF transformable wheel design based on geared 8 bar parallel linkage mechanism

**DOI:** 10.1038/s41598-023-50804-y

**Published:** 2024-01-03

**Authors:** Hyeungyu Yoon, SangGyun Kim, Inha Park, Jaeyeong Heo, Hwa Soo Kim, TaeWon Seo

**Affiliations:** 1 School of Mechanical Engineering, Hanyang University, Seoul, 04763 Republic of Korea; 2 Department of Mechanical Systems Engineering, Kyonggi University, Suwon, 16227 Republic of Korea

**Keywords:** Engineering, Mechanical engineering

## Abstract

This paper introduces a novel design and static optimization for a two-degrees-of-freedom transformable wheel based on a geared linkage mechanism. Overcoming obstacles, including stairs, with small wheels is a major challenge in the field of mobile robotics research. Among various robots, the transformable wheel, which can change the shape of the wheel to overcome steps and optimize the path, was presented and has undergone many improvements. Nevertheless, problems such as asymmetry and structural strength remain. Therefore, the design of this paper aims to address the structural inefficiencies identified in the previous research model, which were attributed to the asymmetric placement of the linear motion guide. Through the implementation of this mechanism, the linear motion of the lobe can be segregated, enabling each input motor to share the workload effectively. The optimization process focus on determining the optimal linkage length under static conditions, resulting in improved structural characteristics and force distribution of linkage within the designated workspace. As a result, asymmetry of motion is eliminated, required intervention angle of the driving motor and stress of linkage was reduced by 36.24% and 8.35%, respectively.

## Introduction

Wheels are highly efficient machines for traveling on level terrain and inclines. Because the wheel surfaces are equidistant from the axle, vehicles and robots employing wheels to navigate smooth terrain can minimize energy wastage. Furthermore, they possess the capability to surmount obstacles smaller than their wheel radius, provided there is adequate frictional force and torque. Conversely, overcoming obstacles larger than the wheel radius proves challenging, necessitating additional devices. Our surroundings feature numerous structures designed for human mobility, including stairs, which pose challenges for wheel-based devices within such facilities.

Expanding the diameter of the wheel to address these challenges introduces spatial inefficiencies. If the height of indoor stairs is *r*, to overcome this with wheels, a minimum diameter of 2*r* is required. In this context, there were attempts to climb stairs using dynamic methods or additional mechanisms while using fixed wheels^1,2^. Considering the wheel that have to overcome the step with slow speed to avoid impact, it become static problem. It requires the theoretical maximum static friction coefficient of 1 and the condition that the edge of the stairs should meet with wheel at a tangential angle of 45°, so the actual wheel size should be at least 6.85*r*. When the height of stair is 0.2 m, the wheel diameter should be 1.37 m and it is not an appropriate size for a mobile service robot platform that moves around people. Therefore, various studies have endeavored to enhance obstacle-handling capabilities relative to wheel diameter, increasing the diameter only when necessary. These studies explore mechanisms such as the origami structure wheel that can change diameter^3–5^, and compliant variable diameter wheels^6^. Additionally, there are several robot that change the shape of wheel. For example, robots like RHex, Loper, and IONS employ wheel-legged mechanisms, altering wheel rims into legs to surmount obstacles^7–9^. A soft robot mechanism that copes with obstacles has also been developed by using shape memory alloy to change the shape of the wheel through electrical signals^10^. Several robots, including Turboquad, have been designed with 1 DOF transformable wheels^11–13^. Moreover, passive mechanisms for shape transformation exist^14,15^. These designs aim to reduce the necessary frictional force to overcome obstacles.

Building upon this research, fixed shapes optimized for specific stair sizes have also been explored^16–19^. Previous research models incorporate arc-shaped spokes for stair climbing. These wheels’ strength lies in their ability to provide an even smoother trajectory compared to other robots, without requiring complex structures. As an extension of this research, research was also conducted on vaious mobile robot platform that could stably climb stairs while applying spoke wheels^20–22^. However, these fixed shapes, lacking circular features, are not well-suited for driving on flat surfaces. Research aiming to combine flat surface driving and step climbing into one passive DOF wheel^23^ yields efficient functionality but sacrifices path smoothness.

The 2-degrees-of-freedom (2-DOF) transformable wheel, named STEP, capable of overcoming various-sized obstacles without the need for additional mechanisms on the target platform, was proposed^24^. This mechanism divides the rim of the wheel into 3 parts and moves with 2 degrees of freedom, and has the advantage of being able to actively respond by modifying the shape according to the size of the stairs. Research on trajectory planning with this mechanism defined the wheel diameter and motion range for various stair sizes^25^. However, this mechanism has a structure in which the mechanism for changing the shape of the wheel protrudes long in the axis direction of the wheel. Because of this, space limitations occurred when applied to a robot platform, and there was a problem of requiring a wheel axle with higher strength. To approach this problem, a wheel design with a modular 7 bar linkage transformable wheel mechanism is presented^26^. This wheel mechanism was reduced in size by using internal rotating linkage instead of protruding features on both sides of the wheel. This mechanism was optimized in terms of linkage length to enhance manipulability and output torque^27^. However, this mechanism exhibits certain inconvenient characteristics during shape changes.

The 7-bar mechanism of STEP consists of seven rotational joints, including two input motors, and possesses three output DOF. To constrain the additional DOF, a prismatic joint was added, effectively reducing the system to 2 DOF output. This prismatic joint was implemented as an linear motion guide but could not be placed at the center of the rim to avoid interference issues^26^. The mechanism inherently exhibits an asymmetric architecture, limiting workspace and input torque distribution. Additionally, the asymmetric motion feature poses challenges for control based on the *r*-$\theta$ model, requiring the intervention of a driving motor to compensate for lobe movement^28^. In summary, the current 7 bar transformable wheel mechanism inherently has asymmetry and workspace limitations due to the linear guide. As a result, this clearly reveals an uncomfortable relationship in which improving any one of structural strength, workspace, and manipulability during the optimization process greatly limits the others^27^.

This paper proposes a new design that eliminates the need for a prismatic joint which limits the workspace and characteristic of motion, addressing also structural weaknesses when torque is applied. A transmission mechanism similar to a differential gear was adopted to enable one-directional linear motion with a single motor input. The other motor input controls the angle of the lobe. By separating these tasks, modifying torque distribution within the workspace becomes much easier, resulting in eliminated asymmetry and reduced strength requirements for the mechanism up to 12.12% and fulfilling the ability to cover entire workspace required. This study includes kinematic analysis of the mechanism and static optimization for a given workspace. This mechanism, an improvement on the 2-DOF transformable wheel, provides the possibility for the mobile robotic platform to move freely on terrain optimized for the human leg. Therefore, this mechanism can be applied to robots operating in indoor environments, such as service robots or transportation robots inside factories.

## Geared 8-bar parallel linkage mechanism for 2-DOF transformation

**Figure 1:**
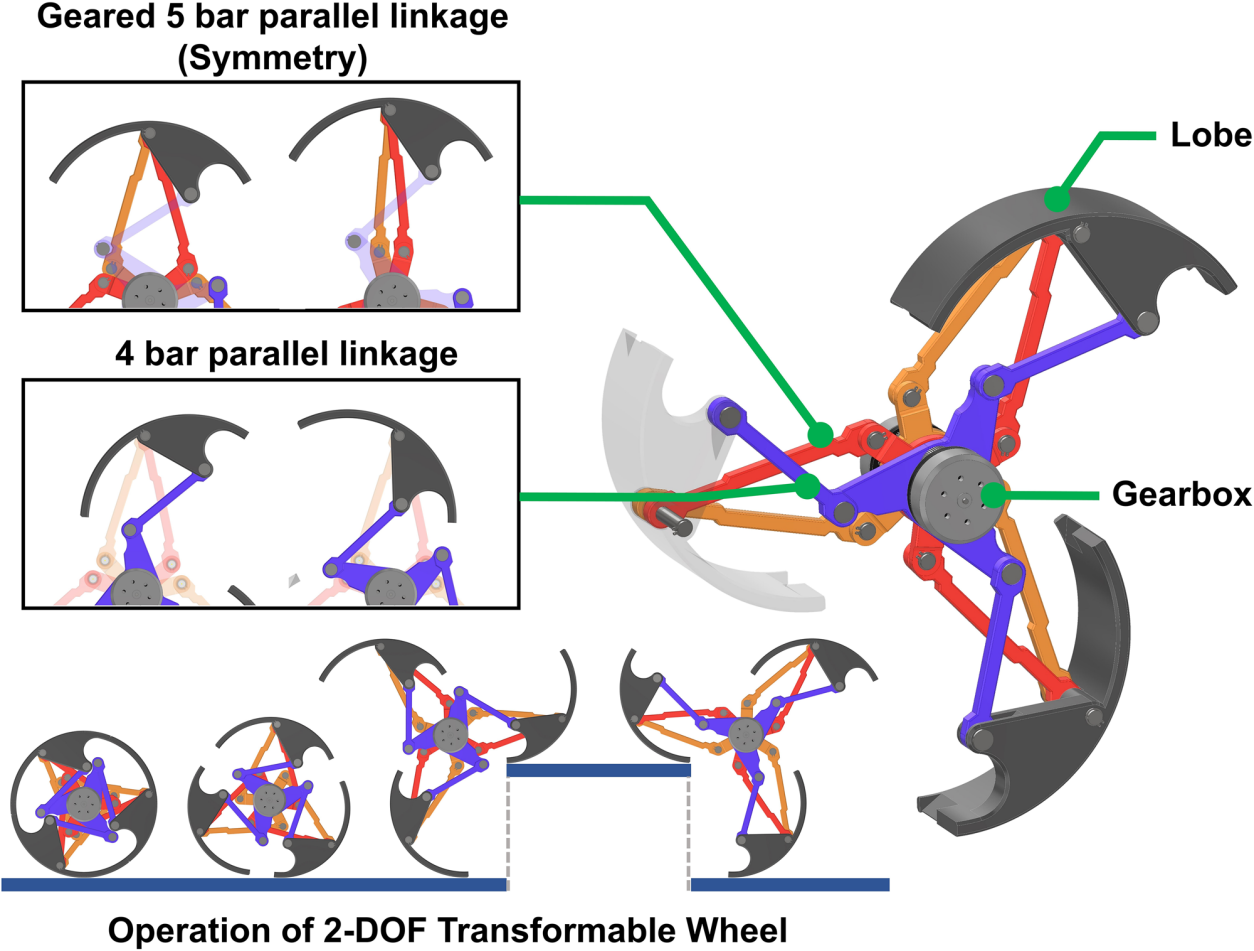
The concept and configuration of 2-DOF 8-bar parallel linkage mechanism applied to the wheel. The overall configuration of the mechanism (Right), Relationship between the movement of each parallel linkage and the position of the lobe (Top left), the operation scenario while overcoming step describing ground and obstacle surface with blue line (Bottom left).

The mechanism for wheel transformation is illustrated in Fig. 1. Each wheel comprises three lobes, 18 linkages, and a central gear. The 8-bar mechanism employed for each lobe comprises a combination of 5-bar (in red and orange) and 4-bar (in blue) parallel links, enabling linear and rotary motion, respectively.

The 5-bar linkage for linear motion inherently possesses 2-DOF inputs and outputs. However, when combined with a gear mechanism to constrain motion between the two input links, it can be transformed into a 1-DOF system for both inputs and outputs. The gear mechanism ensures that each input linkage rotates symmetrically, with the length elements maintaining symmetry as well. Consequently, the center links move exclusively in straight lines. The 4-bar linkage for rotary motion utilizes the output length of the 5-bar linkage on one side, replacing one bar to create a total 8-bar mechanism.

**Figure 2:**
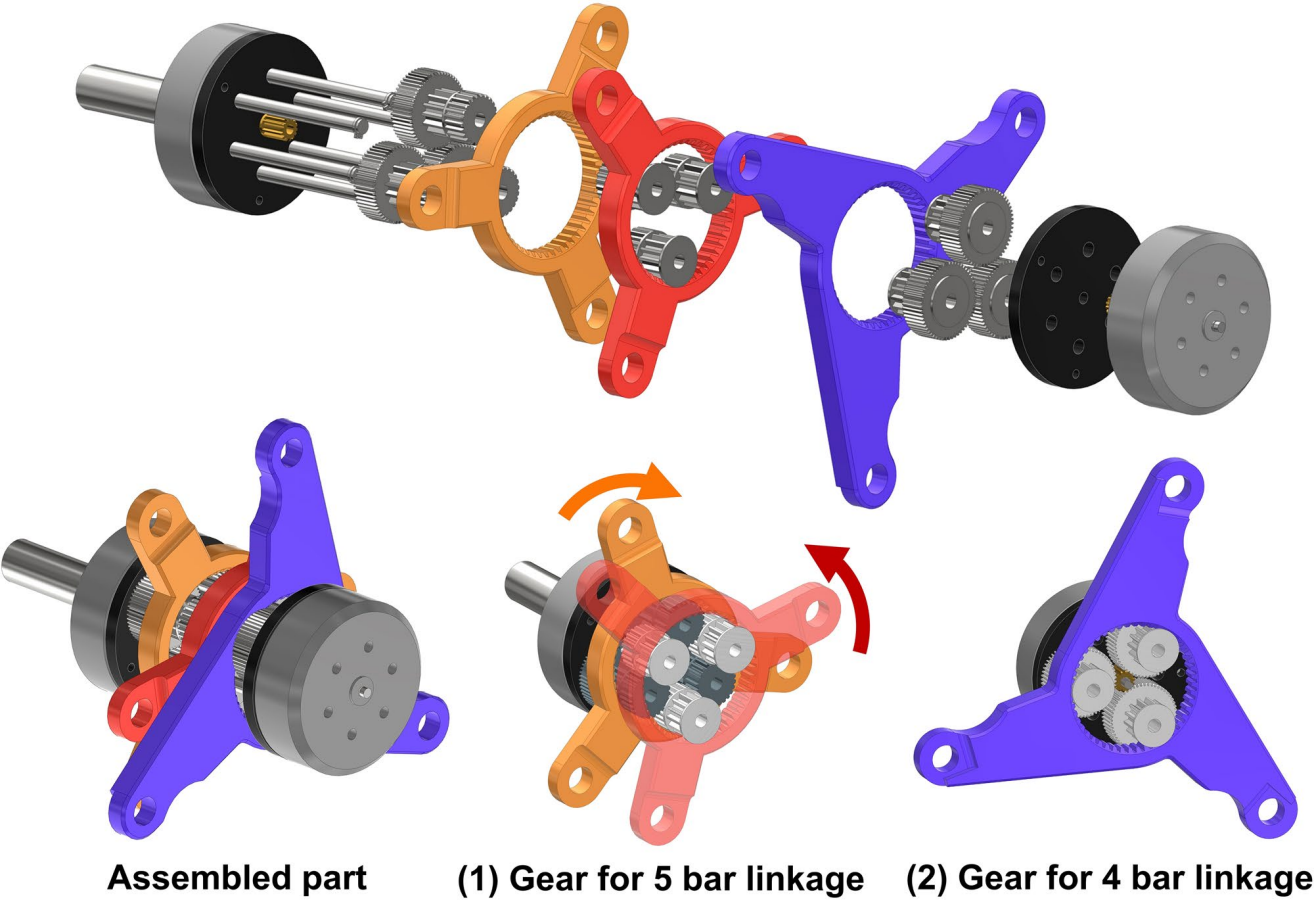
3D CAD design of gearbox and input linkage for the mechanism and its exploded view. (1) The gear for 5 bar linkage receives power from one motor and through deceleration creates the motion of the input linkage with two opposite directions. (2) Another one for 4 bar, which is similar to that of 5 bar, but it has no duplicating opposite direction.

To implement this concept, a gear mechanism is necessary to convert the two motor inputs into three outputs. The gear structure enabling this transformation is depicted in Fig. 2, where one motor is linked to a gear that, upon switching, synchronizes the two input links to move at the same speed but in opposite directions, while the other motor is connected to an independent input link responsible for rotating the lobes. This gear structure is meticulously designed to minimize interference among each link.

**Figure 3:**
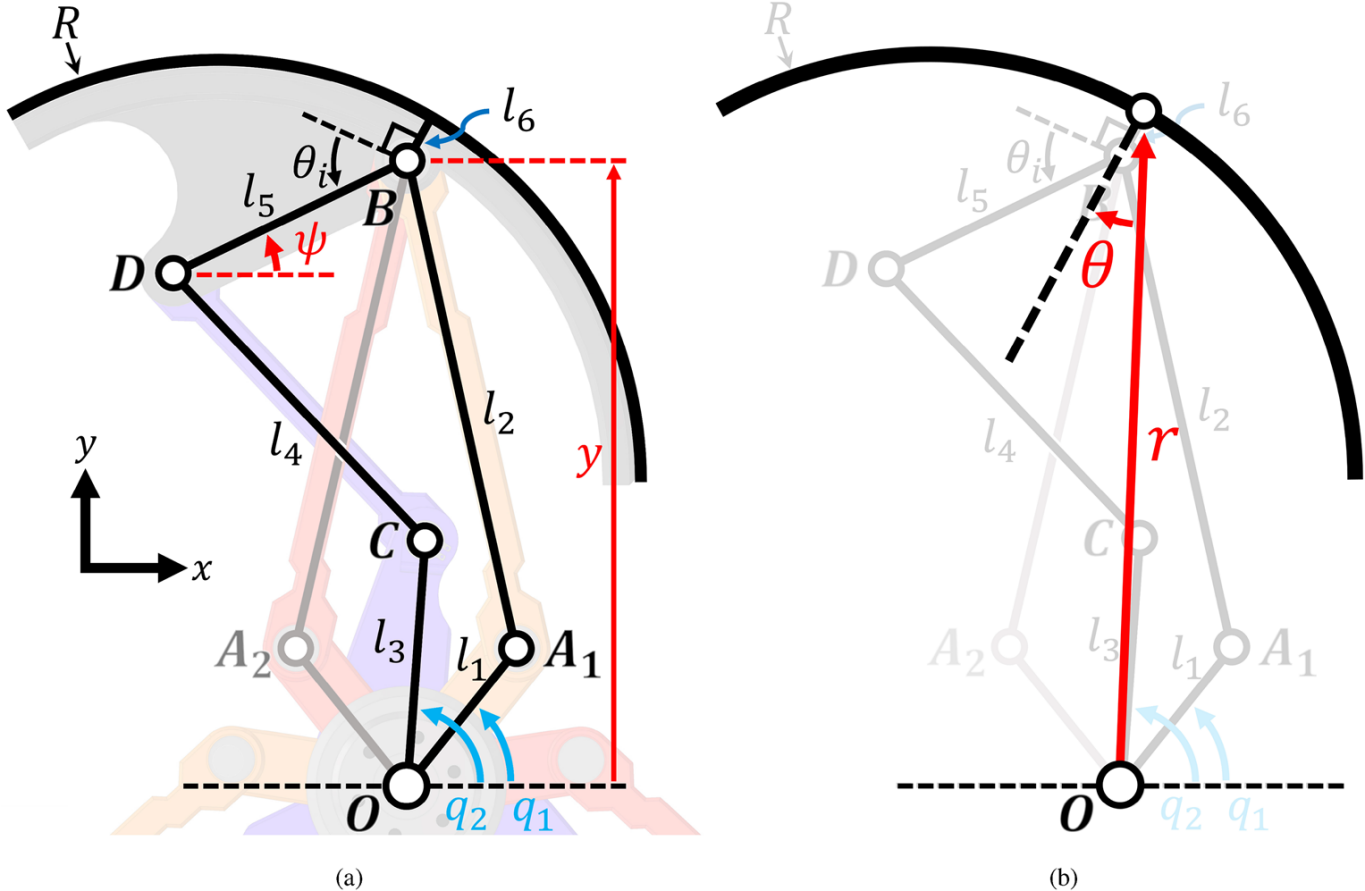
Schematic diagram of kinematics analysis of geared 8-bar parallel linkage mechanism to ascertain the coordinates of all points: (**a**) y and $\psi$, and (**b**) r and $\theta$. One side of the symmetric parallel link is grayed out for visual clarity of the remaining elements. In all the kinematic schematics, inputs are expressed in blue and outputs in red.

Figure 3a depicts the schematic diagram of the 8-bar parallel linkage, illustrating the input variables ($q_1$, $q_2$) and the output variables ($\psi$, *y*) for kinematic analysis. Additionally, this 2-DOF deformation can be expressed in terms of the distance (*r*) between the wheel center and the lobe center and the angle ($\theta$) that the lobe forms with the radial direction of the wheel, as shown in Fig. 3b. The value of *r* can be modified by rotating $q_1$, the input angle of the 5-bar parallel link, while $\theta$ can be controlled by rotating $q_2$, the input angle of the 4-bar parallel link. However, since one side of the 4-bar link responsible for lobe rotation depends on the position of the 5-bar link, changing *r* without altering the lobe’s angle will result in a rotation of $q_2$. Conversely, changing $\theta$ while keeping *r* fixed only necessitates manipulation of $q_2$. The conversion between the $r-\theta$ and $y-\psi$ models can be calculated using the Eqs. (1)–4.

1 $$\begin{aligned} r&=\sqrt{{l_6}^2+y^2+2yl_6\cos {\psi }} \end{aligned}$$

2 $$\begin{aligned} \theta&= \psi -\arccos {\left(\frac{y+l_6\cos {\psi }}{\sqrt{{l_6}^2+y^2+2yl_6\cos {\psi }}}\right)}\end{aligned}$$

3 $$\begin{aligned} y&=\sqrt{{l_6}^2+r^2-2rl_6\cos {\psi }}\end{aligned}$$

4 $$\begin{aligned} \psi&= \theta +\arccos {\left(\frac{r-l_6\cos {\theta }}{\sqrt{{l_6}^2+r^2-2rl_6\cos {\theta }}}\right)} \end{aligned}$$

The center of the lobe is located at point *B* and is at a distance of $l_6$ from the center of the circular (lobe-shaped) wheel. The lobe and the end effector linkage $\overline{DB}$ are interconnected and can be treated as a single rigid body. Therefore, it is more efficient to analyze only the end effector, excluding the lobe, until the static force analysis is conducted.

## Kinematic and static analysis

### Forward and inverse kinematics with geometry method

Kinematic analysis plays a crucial role in determining a mechanism’s workspace and identifying singularities, providing essential information for control. The mechanism under study combines two types of parallel links on a single plane. Analyzing the kinematics of a 2D parallel machine requires constraints to indicate that the links are closed, which can be addressed through analytical or numerical methods. Analytically, constraints can be solved by deriving equations that express the constraints in terms of the coordinates of each joint on a coordinate or complex plane. However, these equations can become challenging to solve analytically when they involve numerous variables or yield multiple roots. Numerical methods are typically employed to address such situations, with the Newton–Raphson method being the most common choice, as it leverages derivatives to find approximate solutions. A previous research model^26^ serves as an example where a numerical solution was used due to the analytical complexities arising from the model’s inclusion of a prismatic joint and a substantial number of terms and roots in the equation^27^. However, models requiring numerical solutions can pose challenges for real-time control due to the extensive computational demands. Another approach is the geometric method, which is explored in this study due to its intuitive nature and the advantage it offers in selecting an appropriate solution from among multiple roots.

To begin, let *O* represent the origin of this mechanism on the coordinate plane. Each point can be expressed using Eqs. (5)–(9).

5 $$\begin{aligned} A_1&=({l_1\cos {q_1}}, {l_1\sin {q_1}}), \end{aligned}$$

6 $$\begin{aligned} A_2&=({-l_1\cos {q_1}}, {l_1\sin {q_1}}), \end{aligned}$$

7 $$\begin{aligned} B&=(0, y),\end{aligned}$$

8 $$\begin{aligned} C&= ({l_3\cos {q_2}}, {l_3\sin {q_2}}),\end{aligned}$$

9 $$\begin{aligned} D&= (-l_5\cos {\psi }, y-l_5\sin {\psi }). \end{aligned}$$

**Figure 4:**
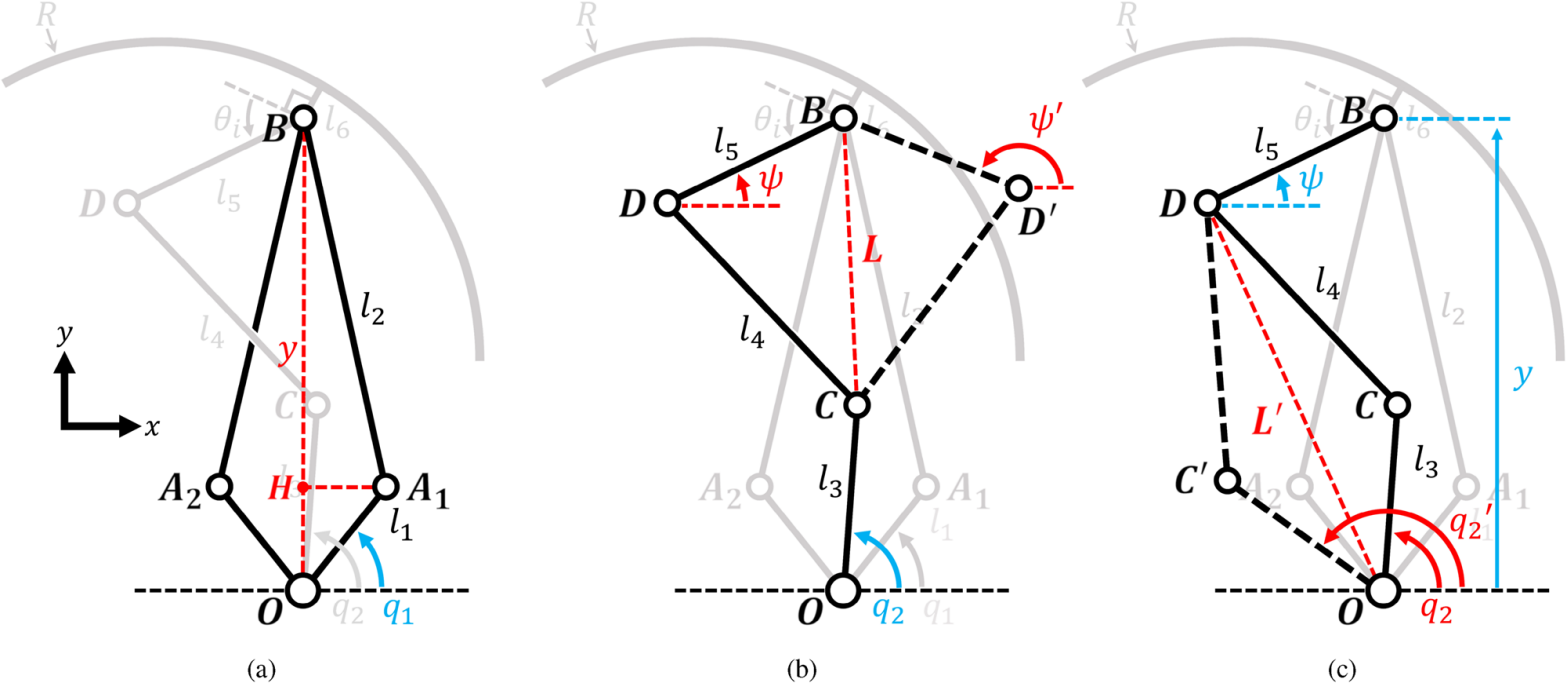
Schematic diagram of forward kinematics analysis at (**a**) geared 5-bar parallel linkage and (**b**) 4-bar parallel linkage mechanism. (**c**) The diagram for inverse kinematic at 4-bar parallel linkage. Other linkages that are not used are grayed out for visual clarity of the remaining elements.

The process of determining constraints for a geared 5-bar parallel link is illustrated in Fig. 4a. Within this figure, $l_1$ and $l_2$ denote the lengths of the elements composing the geared 5-bar parallel link. To initiate the analysis, draw a perpendicular line from point $A_1$ to the center line, and denote the intersection as point *H*. As previously mentioned, $q_1$ represents the input linkage angle of the 5-bar parallel link, while point *B* signifies the position of the end effector determined by this link. The length of line segment $\overline{HO}$ can be determined using trigonometric methods, the length of $\overline{HB}$ can be calculated using the Pythagorean Rule, and by adding them together, we obtain the value of *y*. Eq. (10) provides the expression for *y*, derived through the geometric method.

10 $$\begin{aligned} y=l_1\sin {q_1}+\sqrt{{l_2}^2-{l_1}^2\cos ^2{q_1}}. \end{aligned}$$

Geometric derivations for the four-bar parallel links responsible for rotating the lobes can also be established. Typically, a four-bar linkage possesses no more than two real roots. When there is just one real root, the linkage is considered singular. In cases with two real roots, they exhibit symmetric geometric properties concerning the singularity. Given these principles, in Fig. 4b, points *D* and $D'$ represent the locations determined by these two real roots within the linkage. Within this context, $l_3$, $l_4$, and $l_5$ correspond to the lengths of the elements comprising the 4-bar parallel linkage. To proceed, draw a straight line connecting points *B* and *C*, and calculate its length, denoted as *L*, using the Euclidean distance formula (Eq. 11). Subsequently, the cosine law can be applied to determine the angle $\angle {DBC}$ within the $\triangle {DBC}$ (Eq. 12). If this triangle satisfies the condition where the length of one side is less than the sum of the lengths of the other two sides, then angle $\alpha _F$ ($\angle {DBC}$) must exist as a value ranging from 0 to $\pi$ (rad). The angle $\beta _F$ ($\angle {OBC}$) can be calculated from the coordinates of point B and C, which are components of the vector ${\overrightarrow{BC}}$, through inverse tangent calculations within the appropriate quadrants (Eq. 13). Both $\psi _1$ and $\psi _2$ can be expressed as the sum and difference of alpha and beta relative to a vertical line extending upward from the origin, as depicted in Eqs. (14) and (15).

11 $$\begin{aligned} L&= \parallel {\overrightarrow{BC}}\parallel \end{aligned}$$

12 $$\begin{aligned} \alpha _F&= \arccos \left( {\frac{{l_5}^2+{L}^2-{l_4^2}}{2l_5L}}\right) \end{aligned}$$

13 $$\begin{aligned} \beta _F&= \arctan {\left( \frac{{l_3\cos {q_2}}}{y-{l_3\sin {q_2}}}\right) } \end{aligned}$$

14 $$\begin{aligned} \psi&=\frac{\pi }{2}-\alpha _F+\beta _F \end{aligned}$$

15 $$\begin{aligned} \psi '&=\frac{\pi }{2}+\alpha _F+\beta _F \end{aligned}$$

When provided with *y* and $\psi$, the inverse kinematics for determining $q_1$ and $q_2$ that satisfy these values can also be derived employing the same geometric method as previously described. This involves drawing a straight line $L'$ from point *D* to point *O*, as shown at Fig. 4c. The outcomes are provided by Eqs. (16)–(18) below.

16 $$\begin{aligned} q_1&= \arcsin {\left( \frac{{l_1}+y^2-{l_2}^2}{2yl_1}\right) } \end{aligned}$$

17 $$\begin{aligned} q_2&= \frac{\pi }{2}+\alpha _I+\beta _I \end{aligned}$$

18 $$\begin{aligned} q_2'&= \frac{\pi }{2}-\alpha _I+\beta _I \end{aligned}$$

where

19 $$\begin{aligned} {\alpha _I=\arccos \left( {\frac{{L'}^2+{l_3}^2-{l_4^2}}{2l_3L'}}\right) }, {\beta _I = \arctan {\left( \frac{l_5\cos {\psi }}{y-l_5\sin {\psi }}\right) }} \end{aligned}$$

### Jacobian and static force analysis

The Jacobian is driven by the time derivative of the end effector position *y* and constraint equations. The velocity of end effector is shown in Eq. (20).

20 $$\begin{aligned} \dot{y}=\left( l_1\cos {q_1}-\frac{{l_1}^2\cos {q_1}\sin {q_1}}{\sqrt{{l_2}^2-{l_1}^2}\cos ^2{q_1}}\right) \cdot {\dot{q_1}} \end{aligned}$$

The constraint equation can be formulated based on the length condition. To satisfy this condition, the distance between points *D* and *C* must equal $l_4$, which can be represented using Eq. (21).

21 $$\begin{aligned} (l_3\cos {q_2}+l_5\cos {\psi })^2+(l_3\sin {q_2}+l_5\sin {\psi }-y)^2-{l_2}^2=0 \end{aligned}$$

Taking the partial derivative of this expression yields Eq. (22), with coefficients denoted as $G_1$, $G_2$, and $G_3$, which are functions of $q_1$ and $q_2$.

22 $$\begin{aligned} 0&= G_1(q_1, q_2)\cdot \dot{q_1}+G_2(q_1, q_2)\cdot \dot{q_2}+G_3(q_1, q_2)\cdot {\dot{\psi }} \end{aligned}$$

The Jacobian is represented by Eq. (23), and all the elements of the Jacobian are computed as shown in Eqs. (24)–(26).

23 $$\begin{aligned} J&= \begin{bmatrix} Y &{} 0 \\ \Gamma _1 &{} \Gamma _2 \end{bmatrix} \end{aligned}$$

where

24 $$\begin{aligned} \Gamma _1&= -\frac{G_1}{G_3} \end{aligned}$$

25 $$\begin{aligned} \Gamma _2&= -\frac{G_2}{G_3} \end{aligned}$$

26 $$\begin{aligned} Y&= l_1\cos {q_1}-\frac{{l_1}^2\cos {q_1}\sin {q_1}}{\sqrt{{l_2}^2-{l_1}^2}\cos ^2{q_1}} \end{aligned}$$

The Jacobian is related to the velocity of the end effector, $\dot{y}$ and ${\dot{\psi }}$, as well as the torque applied to the input motors, $\tau _1$ and $\tau _2$, as demonstrated in Eqs. (27) and (28). Here, *F* and *T* represent the force and torque applied to the end effector. Additionally, the Jacobian is employed to assess manipulability.

27 $$\begin{aligned} \begin{bmatrix}\dot{y} \\ {\dot{\psi }} \end{bmatrix}&= J\cdot \begin{bmatrix}\dot{q_1} \\ \dot{q_2} \end{bmatrix} \end{aligned}$$

28 $$\begin{aligned} \begin{bmatrix}\tau _1 \\ \tau _2 \end{bmatrix}&= J^T\cdot \begin{bmatrix}F \\ T \end{bmatrix} \end{aligned}$$

**Figure 5:**
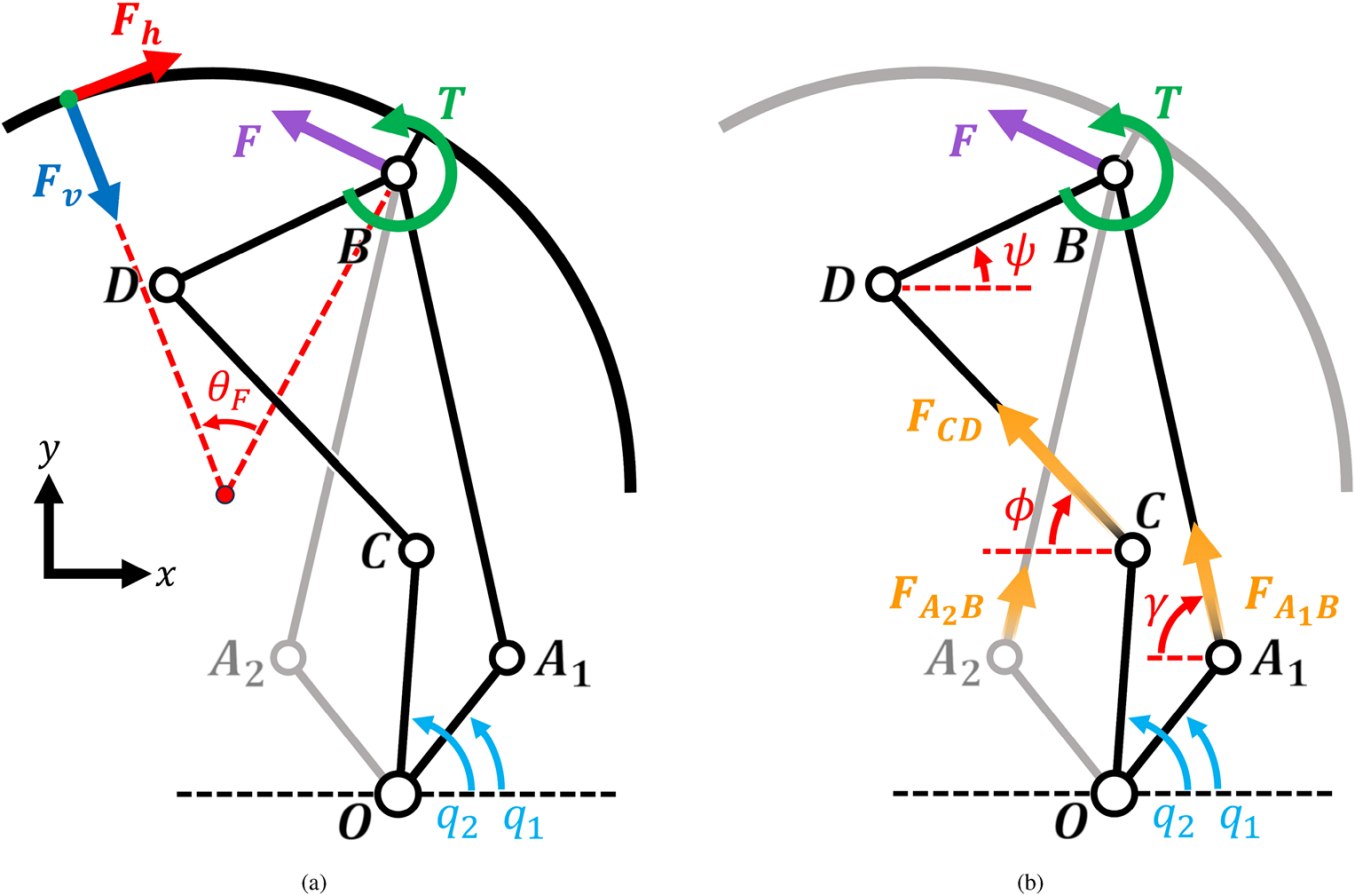
Schematic diagram of Static analysis showing equilibrium between the force at the lobe and the force and torque from end effector (**a**), and between the force and torque from end effector and linkage force (**b**).

To assess the structural integrity of the linkage mechanism, it is necessary to calculate the forces acting on each linkage component. When the wheel is in motion, forces of varying magnitude and direction are exerted on the lobes. Figure 5a illustrates the equilibrium between the force at the lobe and the force with torque from the end effector. The input force applied to a lobe can be categorized into two components: those perpendicular ($F_v$) to its tangent and those horizontal ($F_h$). The point at which this force is applied can be defined by an angle $\theta _F$ measured from the lobe center. The direction and location of the force with respect to the end effector depend on the tilt of the lobe ($\theta$). Therefore, the forces and torques that act on the end effector ($F_{x1}$, $F_{y1}$ and *T*) to maintain equilibrium can be expressed using Eqs. (29) and (30).

29 $$\begin{aligned} F_{x1}&= F_v(R-l_6)\sin {(\theta _F+\theta )}+F_h\cos {(\theta _F+\theta )} \end{aligned}$$

30 $$\begin{aligned} F_{y1}&= -F_v(R-l_6)\cos {(\theta _F+\theta )}+F_h\sin {(\theta _F+\theta )}) \end{aligned}$$

31 $$\begin{aligned} T&= F_v(R-l_6)\sin {\theta _F}-F_h(R-(R-l_6)\cos {\theta _F}) \end{aligned}$$

The structural strength is contingent upon the forces acting on linkages *CD*, $A_1B$, and $A_2B$, as depicted in Fig. 5b. Since the torque and force applied to the input linkages ($OA_1$, $OA_2$, *OB*) and the lobe (*BD*) are determined by the forces within these aforementioned linkages, these three linkages can represent the structural strength. Moreover, they encompass torque, which cannot be directly compared to the force in the objective function for optimization due to differences in dimension. The force acting on linkage *CD* can be derived from the torque *T* and is expressed as shown in Eq. (32).

32 $$\begin{aligned} F_{CD}&= \frac{T}{\sin {(\psi +\phi )}} \end{aligned}$$

The forces acting on the geared 5-bar parallel linkage ($F_{x2}$, $F_{y2}$) are determined by subtracting $F_{CD}$ from $F_{x1}$ and $F_{y1}$, as illustrated in Eqs. (33) and (34). To calculate the forces acting on linkages $A_1B$ and $A_2B$, simultaneous equations are solved, as demonstrated in Eqs. (35) and (36).

33 $$\begin{aligned} F_{x2}&= F_{x1}+F_{CD}\cos {\phi } \end{aligned}$$

34 $$\begin{aligned} F_{y2}&= F_{y1}-F_{CD}\sin {\phi } \end{aligned}$$

35 $$\begin{aligned} F_{A_1B}&= \frac{1}{2}\left( \frac{F_{y2}}{\sin {\gamma }}+\frac{F_{x2}}{\cos {\gamma }}\right) \end{aligned}$$

36 $$\begin{aligned} F_{A_1B}&= \frac{1}{2}\left( \frac{F_{y2}}{\sin {\gamma }}-\frac{F_{x2}}{\cos {\gamma }}\right) \end{aligned}$$

## Optimization of linkage with genetic algorithm

The objective of the optimization process is to enhance the structural integrity of the system, enabling it to maintain its shape under various conditions. The forces acting on the linkages representing structural strength were derived in the previous section ($F_{CD}$, $F_{A_1B}$, $F_{A_2B}$).

The transformable wheel incorporates two parallel linkages to create an 8-bar parallel linkage mechanism. Given the inherent complexity of parallel linkages, they exhibit intricate characteristics in terms of length relationships and maneuverability. Specifically, for a given wheel shape, it may not always be feasible to achieve that shape, or the path taken to attain that shape may not constitute the shortest distance. This signifies that the mechanism’s characteristic with regard to linkage length is neither continuous nor differentiable, a condition that poses challenges for most optimization algorithms requiring differentiability.

In such scenarios, genetic algorithms can provide a viable solution. As a type of heuristic algorithm, genetic algorithms offer the advantage of seeking a global optimum without demanding continuity and differentiability.

### Design variables and object function

The shape and characteristics of this transformable wheel are determined by the seven variables depicted in the schematic above. Through analysis and adjustment of the size of each variable, it was determined that all of these variables contribute to the mechanism’s characteristics. Consequently, the design variables were established as the seven variables presented in Table 1. These variables are bounded by lower bounds (lb) and upper bounds (ub), which are determined by the wheel size. For instance, $l_1$ and $l_2$ have lower bounds of 0.03 m because they must exceed the radius of the gearbox. The wheel’s radius, *R*, is set at 0.125 m, consistent with the dimensions used in a previous study model^24^.

**Table 1:** Variables used in genetic algorithm optimization.

Variables (Genes)	$l_1$(m)	$l_2$(m)	$l_3$(m)	$l_4$(m)	$l_5$(m)	$l_6$(m)	$\theta _i(deg)$
Lower bound	0.03	0.00	0.03	0.00	0.00	0.00	$-180$
Upper bound	R	2R	R	2R	R	R	180

The upper and lower bounds at length are set as the maximum range that can exist between the gearbox radius (0.03 m) and wheel radius *R*.

The optimization process necessitates constraints to ensure that the results remain feasible for implementation, such as preventing the linkage from protruding through the lobe. To restrict the motion range of the linkage, it is possible to monitor the positions of points $A_1$, $A_2$, *C*, and *D* to ensure they meet specific conditions when calculating the inverse kinematics for the objective function.

The objective function must encompass structural strength across the entire workspace and the range of input forces on the lobe. Consequently, the function was designed to consider all scenarios: (1) for every wheel shape within the workspace, and (2) for every contact force that the ground can exert on the lobe. With seven design variables, including $q_1$ and $q_2$ for situation (1), and $\theta _F$, $F_v$, and $F_h$ for situation (2), it is used to calculate static forces. By setting $F_v$ to 1, we can determine that the maximum value of $F_h$ should be 1 (representing the theoretical maximum friction coefficient), allowing for a direct assessment of the increase/decrease ratio of the force acting on the linkage. The situation variable vector $s=[\theta _F, q_1, q_2]$ is composed of combinations of elements from $\theta _F$, with 31 equally spaced samples ranging from $-60$ degrees to 60°, and $q_1$ and $q_2$ with 41 samples calculated using inverse kinematics from the workspace in accordance with equally spaced stair sizes. The relationship between stair size and the workspace of the transformable wheel is defined in previous research^24^. The thread length (*T*) spans from 0.26 to 0.34 m, while the height (*H*) ranges from 0 to 0.2 m. Consequently, it is essential to define the relationship between stair size and the shape of the $r-\theta$ model as Eqs. (37) and (38).

37 $$\begin{aligned} T&= R\left( \frac{2\pi }{3}-2\sin {\frac{\pi }{3}}\right) +r\left( \cos {\left( \theta +\frac{\pi }{6}\right) }+\sin {\left( \theta +\frac{\pi }{3}\right) }\right) \end{aligned}$$

38 $$\begin{aligned} S&= r\left( \sin {\left( \theta +\frac{\pi }{6}\right) }-\cos {\left( \theta +\frac{\pi }{3}\right) }\right) \end{aligned}$$

The number of samples is determined with consideration for computational time and resolution. The combinations of design variables are represented as the vector $x=[l_1, l_2, l_3, l_4, l_5, l_6, \theta _i]$.

Thus, the objective function, which calculates the maximum force by comparing all 52,111 ($31\times 41\times 41$) combinations of *s* within the given design variable set *x*, is expressed as Eq. (39).

39 $$\begin{aligned} f_{obj}(x) = \underset{s\subset S^3}{max}[F_{A_1B}(s,x), F_{A_2B}(s,x), F_{CD}(s,x)] \end{aligned}$$

### Optimization

The parameter configurations for GA optimization are presented in Table 2. Given that the linkage lengths are specified in meters as variables, a vector with a double data type is utilized, and each generation comprises 200 individuals. The fitness scaling function, employed as a criterion for selecting parents in the next generation, is set to rank, while adaptive feasibility, often applied to objective functions with constraints, is utilized for mutation. Considering the diverse combination characteristics of the lengths of each linkage, the crossover method that facilitates genetic information mixing is designated as scattered, with a ratio of 0.5 to actively promote information exchange. The optimization process is programmed to terminate after reaching generations where the minimum score has been observed on more than 50 occasions.

**Table 2:** Variables used for genetic algorithm optimization.

GA parameters		
1.	Population type	Double vector
2.	Population size	200
3.	Fitness scaling function	Rank
4.	Selection function	Stochunif
5.	Mutation function	Adapt feasible
6.	Crossover function	Scattered
7.	Crossover fraction	0.5

The upper and lower bounds at length are set to the maximum range that can exist in wheel radius *R*.

In the entire range of linkage lengths, the region where a solution exists is considerably narrower than the region without a solution. If there is any region within the linkage length combination space where no solution exists, the objective function consistently returns a value of 100. Consequently, it is common to determine that convergence has been reached and terminate the optimization process. Therefore, an algorithm was initially required to generate genes within a specific region, which is detailed in Table 3. The initial parameter range is solely employed to generate the first generation of genes and does not influence the mutation and evolution of subsequent generations. To calculate the initial region, the region where a solution exists was deduced by computing all combinations obtained by equally dividing all variables into 7 intervals (resulting in $7^7$ cases).

**Table 3:** The initial upper and lower bounds of variables.

Variables (Genes)	$l_1$(m)	$l_2$(m)	$l_3$(m)	$l_4$(m)	$l_5$(m)	$l_6$(m)	$\theta _i(deg)$
Initial lb	0.03	0.07	0.03	0.09	0.04	0.00	13
Initial ub	0.08	0.20	0.12	0.13	0.09	0.07	68

The bound is obtain from the maximum and minimum value that can cover entire workspace.

## Result and analysis

### Optimized parameter and comparison

The genetic algorithm optimization process concluded after 371 generations when the values converged, yielding the optimized values as displayed in Table 4. The score attained is 6.234, signifying that when the lobe makes contact with the ground and exerts a force *F*(N), the theoretical maximum force acting on the linkage is 6.234*F*(N) in statics.

**Table 4:** The optimized parameters of geared 8 bar linkage mechanism for transformable wheel.

Optimized design parameter							
Variables	$l_1$(m)	$l_2$(m)	$l_3$(m)	$l_4$(m)	$l_5$(m)	$l_6$(m)	$\theta _i$(deg)
Value	0.053	0.150	0.074	0.110	0.078	0.013	56

Various optimization settings were explored, including adjustments to the population size, mutation rate, and selection function. However, these alterations yielded similar optimized values as long as the optimization process did not diverge. This type of problem does not appear to be significantly influenced by detailed settings.

**Figure 6:**
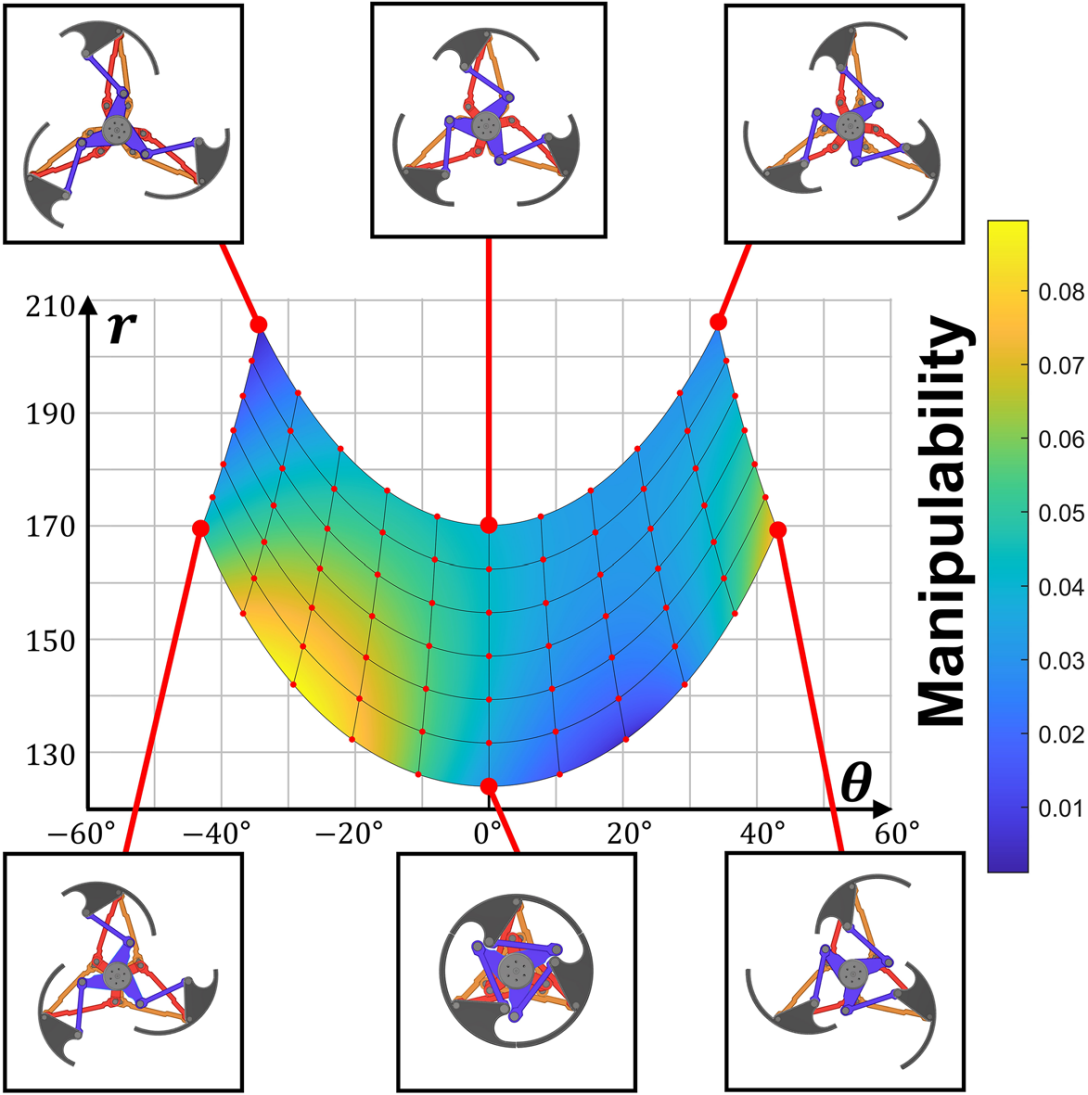
The workspace and manipulability of optimized design. The shape of wheels are marked as supplementary descriptions.

To assess the kinematic characteristics of a particular linkage mechanism, the examination of manipulability is a commonly employed approach. Manipulability indicates the extent to which the end effector’s degree of freedom can be ensured by a specific mechanism in a given posture. In the case of a simple linkage structure, even the required torque can be compared. Manipulability can be calculated using Eq. (40). Figure 6 depicts the workspace with manipulability and the posture of the transformable wheel. In this 2-DOF transformable mechanism, efficient shape changes can be achieved in regions with high manipulability. However, this also implies that high motor torque is required in such regions, while low manipulability regions correspond to lower required torque even at lower speeds. Given that the units for output differ between angle and length, it is preferable to consider the relative value of manipulability in relation to the surrounding values rather than focusing solely on its absolute value.

40 $$\begin{aligned} Manipulability&= \sqrt{det(JJ^T)} \end{aligned}$$

A comparison was made with the current model regarding the limitations of the previous modular 7 bar model mentioned in the introduction of this paper^26,27^ and the result is presented at Table 5. (1) Similarity to the $r-\theta$ model presented in previous control studies, (2) distribution of load received by the wheel during operation, and (3) coverage of the various stair size are the main focus of this paper, so they can be evaluation criteria. Similarity to the $r-\theta$ model can be found by calculating the range in which the center of the lobe moves within the entire workspace. As can be seen in Fig. 3b, as the lobe rotates, the center of the lobe moves and the direction of the *r* vector changes, and this angle range becomes an evaluation index. Additionally, asymmetry refers to the percentage of the difference in the range in which the *r* vector rotates in each direction (Clockwise or Counterclockwise) to the overall range, based on the line connecting the center of the lobe and the center of the wheel when the wheel has a circular shape. Maximum linkage force amplification, which is also used as object function for optimization, refers to the amplification rate when the load applied to the wheel gives maximum force to the linkage. In other words, the situation where linkage receives force in the most disadvantageous direction and position is calculated with statics. Ratio of climbable step size to wheel radius can be calculated by dividing the maximum climbable step size by the wheel radius.

**Table 5:** Performance comparison with previous 7 bar models.

Evaluation indicators	7 bar	7 bar (optimized)	Geared 8 bar
Lobe center movement range (deg)	8.676	7.629	4.864
Asymmetry (percent)	23.32	81.44	0
Maximum linkage force amplification	6.802	7.094	6.234
Ratio of climbable step size to wheel radius	1.20	0.96	1.60

Range of lobe center movement and asymmetry are evaluation factors that can indicate the proximity to the $r-\theta$ model, and maximum linkage force amplitude does the structural strength. There is also indication about ratio climbable step size ratio.

It can be seen that the lobe center movement range has decreased by at least 36.2% compared to previous models. This means that the driving motor has reduced intervention to compensate for the change in lobe center that occurs when the wheel transforms. Additionally, due to the intrinsic properties of the mechanism, the asymmetry of the lobe motion is zero. The maximum linkage force amplification, which determines structural strength, was reduced by at least 8.35%. The ratio of climbable step size to wheel radius may vary in detail depending on the initial design goals, but it can be seen that this mechanism covers more workspace while also providing better results in other metrics. In this comparative analysis, the design of this paper was compared to the initial design of the 7 bar mechanism, as the initial 7 bar mechanism showed the highest performance among the previous models in these evaluation criteria. The optimized 7 bar transformable wheel was at a disadvantage in these evaluation criteria because it was improved with system agility as the objective function, but when compared to the performance of the initial design, a clear trade-off relationship can be seen.

### Additional feature of design

In this study, optimization aimed to derive the most efficient structure of this mechanism while minimizing the inclusion of other design elements. Consequently, a situation arose in which the linkage overlapped between the lobe end and the gearbox. Nevertheless, this issue can be resolved by modifying the shape of the linkage and adjusting the size of the gearbox. In the scenario where the end of the lobe is closest to the center of the wheel within the entire workspace, the distance is approximately 63 mm. Therefore, the diameter of the gearbox was set to 60 mm. Deformation of the linkage was implemented in the area where interference occurred, and the issue was resolved by creating a groove that allowed the linkage to pass through the lobe end without affecting the surface.

## Conclusion

This study introduced a 2-DOF 8-bar transformable wheel mechanism designed for overcoming obstacles, including stairs. The motivation behind this mechanism was to address the asymmetry and workspace limitations observed in previous research models. Furthermore, considering the aim of achieving a lightweight design for suspension and dynamic characteristics, the study encompassed efforts to enhance the structural rigidity of this linkage mechanism. To optimize structural stiffness, the study included a static analysis involving mechanical analysis. Given the inherent discontinuity and non-differentiability of the parallel linkage’s solution characteristics, a genetic algorithm was employed to tackle this challenge. The study also provided an overview of the mechanism’s characteristics across the entire workspace and elucidated the design methodology for practical implementation. It is important to note that the optimization process in this study analyzed only the forces acting on three specific linkages. These forces indirectly determine the forces and torques affecting the other interconnected linkages but do not account for actual stress. Furthermore, the analysis of these forces was conducted solely from a static standpoint. To advance this concept as a follow-up study, dynamic characteristics analysis and more rigorous optimization and design will be pursued.

## Data Availability

All data generated or analysed during this study are included in this published article.
